# Outcomes of one-staged procedures to treat aortic coarctation complicated by cardiac anomalies

**DOI:** 10.1186/s12872-022-02739-x

**Published:** 2022-07-03

**Authors:** Hongyuan Lin, Yi Chang, Xiangyang Qian, Cuntao Yu, Xiaogang Sun

**Affiliations:** Cardiac Surgery Centre, Fuwai Hospital, Chinese Academy of Medical Sciences and Peking Union Medical College, No. 167, North Lishi street, Xicheng District, Beijing, 100037 China

**Keywords:** Aortic coarctation, Bilateral aortofemoral bypass, Hybrid, Complicated COA, One-staged

## Abstract

**Objective:**

One-staged surgical treatment of aortic coarctation combined with cardiac anomalies is challenging. We aim to evaluate the feasibility of bilateral aortofemoral bypass technique in one-staged surgery treating coractation by comparing surgical outcomes with catheter intervention plus stent (hybrid).

**Methods:**

Between January 2012 and December 2017, 50 patients underwent one-staged surgical procedures to treat coarctation and repair concomitant cardiac anomalies, like aortic root dilatation, cardiac valvular disease and so on. Among them, 30 patients underwent bilateral aortofemoral bypass and 20 patients underwent hybrid procedure to treat coarctation. We retrospectively analyzed the data of these patients and compared the early and late results.

**Results:**

All the baseline clinical characteristics were comparable between groups except that the mean age of bypass group was 39.5 ± 14.0 years which was older than hybrid group (27.9 ± 8.5 years, *P* = 0.002). Technical success was achieved in all patients, with no hospital death or other severe complications. Immediately after surgery, in bypass and hybrid group, the mean upper-limb systolic blood pressure decreased from 159.4 to 119.7 mmHg and 148.4 to 111.6 mmHg, the median peak systolic gradient decreased from 68.0 to 10 mmHg and 46.5 to 10 mmHg respectively (*P* = 0.09). And the mean upper-lower limbs gradient decreased from 21.7 to 5.9 mmHg and 21.0 to 2.7 mmHg respectively (*P* = 0.104). The mean follow-up time was 76.92 ± 18.7 in bypass group and 85.4 ± 20.6 months in hybrid group. There were 4 late deaths in bypass group (one died of gastrointestinal bleeding, one died of pulmonary embolism and the other two died of heart failure caused by mechanical prosthetic valve dysfunction). The follow-up peak systolic gradient and other blood pressure parameters showed stable and no differences between two groups.

**Conclusions:**

The bilateral aortofemoral bypass surgery is a safe and effective method which can be used in one-staged surgical strategy to treat coarctation complicated by cardiac anomalies and can be an alternative to the hybrid method.

**Supplementary Information:**

The online version contains supplementary material available at 10.1186/s12872-022-02739-x.

## Introduction

Aortic coarctation (COA) accounts for 5–7% of congenital heart disease [1]. In adults, COA is more likely to be associated with other cardiac anomalies [2], for instance, bicuspid aortic valve, aortic regurgitation or stenosis and ventricular septal defect [2], leading to a challenge for surgeons. The traditional approach is a staged procedure using both median sternotomy and left thoracotomy. However, there is a considerably increased risk when COA repair is performed ahead of severe concomitant cardiac anomalies [3]. Furthermore, damage to the recurrent laryngeal or phrenic nerves, bleeding in long-standing COA with extensive collateral vessels and aneurysms in the treated segment are main deficiencies of surgical correction [4, 5]. In recent years, in order to reduce the risk of staged procedures, one-stage repair for COA combined with other cardiac anomalies has been tried in an increasing number of cardiac centers [6–8]. The most common one-stage strategies for COA management include ascending to descending or abdominal aorta extra-anatomic bypass [7, 9] and hybrid balloon angioplasty with stent implantation [8, 10, 11]. However, there are no reports of one-stage strategy using aortofemoral bypass (AFB), as another extra-anatomic manner, to treat such complicated COAs. Recently, the stenting technique has gradually replaced the traditional surgical correction of isolated COA in adult patients [12], attributing to its avoidance of the acute complications caused by thoracotomy and its satisfactory outcomes [12, 13]. In spite of that, acute aortic wall injury is often a dramatic event of the stenting technique, and may be more frequent in elderly patients with highly calcified tissue or hypoplastic vessels with reduced elasticity [14]. In contrast, extra-anatomic bypass could be feasible in all candidates irrespective of COA features. Theoretically, among extra-anatomic strategies, AFB is an easy and simple method with minimal trauma and lowest risk. The objective of this study is to evaluate the feasibility of AFB technique in one-staged surgery treating COA by comparing surgical outcomes with hybrid technique.

## Patients and methods

This study had been approved by institutional review board (IRB) of Fuwai hospital, Peking union medical college and Chinese academy of medical sciences. All the consents of patients had been obtained. The IRB approval number is 2020–1402, the date is November 24, 2020.

**Fig.1:**
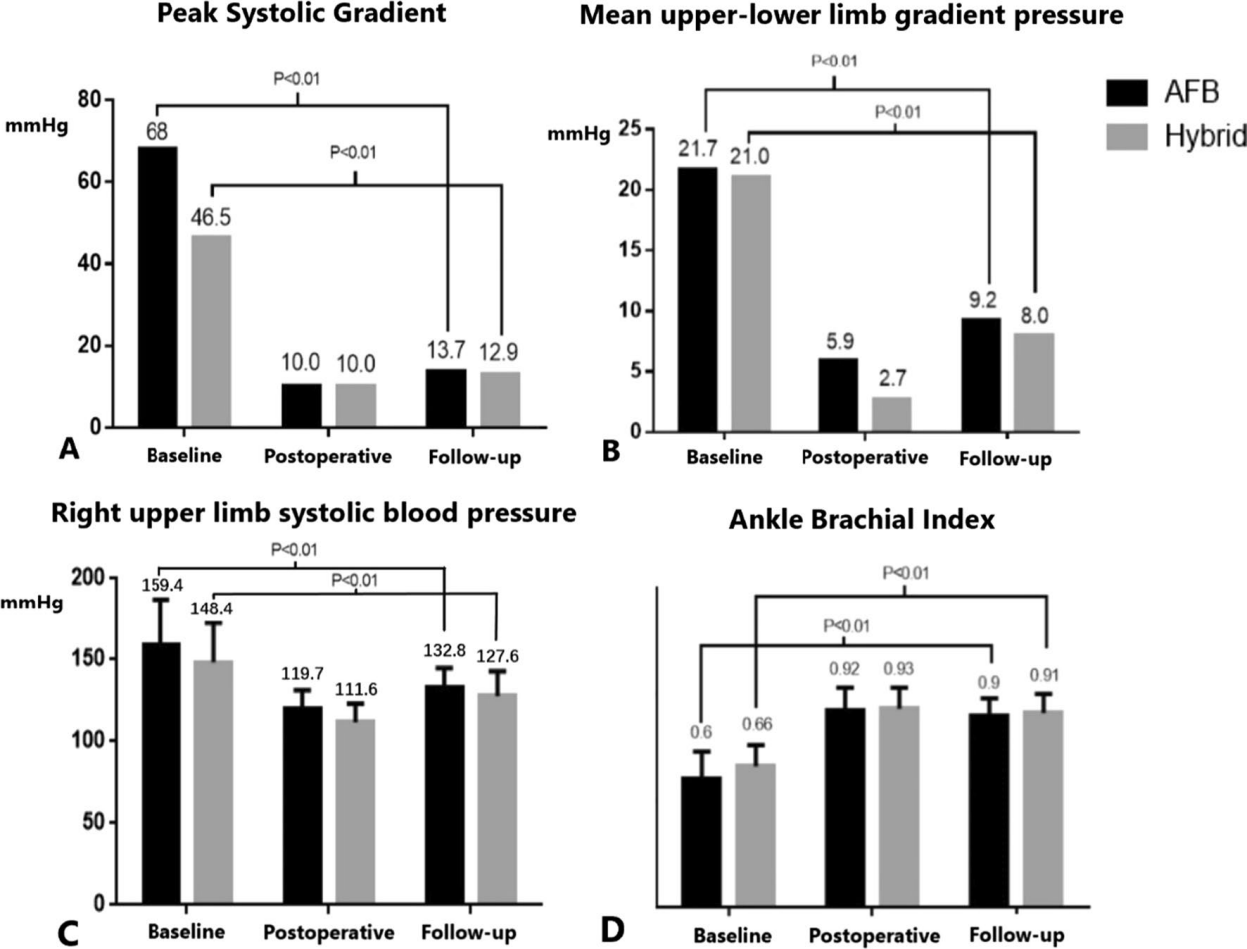
Comparison of blood pressure related parameters between groups

Between January 2012 and December 2017, we enrolled consecutive COA patients who were concomitant with other cardiac anomalies underwent one-stage AFB surgery or one-stage hybrid surgery using femoral pathway. Patients eligible for AFB or stent implantation had native COA associated with hypertension (right upper limb’s non-invasive blood pressure > 130/80 mmHg) and a resting non-invasive pressure gradient > 20 mmHg between upper and lower limbs [15]. Excluded patients had one of the following: (1) age < 16 years; (2) contraindication to antiplatelet or anticoagulation therapy; (3) other etiology of secondary hypertension. All patients were preoperatively examined by echocardiography and aortic computed tomography angiography (CTA) to assess cardiac structural and functional abnormalities and severity of aortic coarctation. In this study, we exhibited the severity of coarctation using the ratio (%) of the narrowest COA dimension (mm) to the dimension of the normal descending aorta at diaphragm level (mm) [16]. The upper and lower limb blood pressures (BPs) for analysis were non-invasively measured in all patients. The upper limb BPs were measured in the right side (Fig. 1).

### Surgical technique

#### AFB strategy

**Fig. 2:**
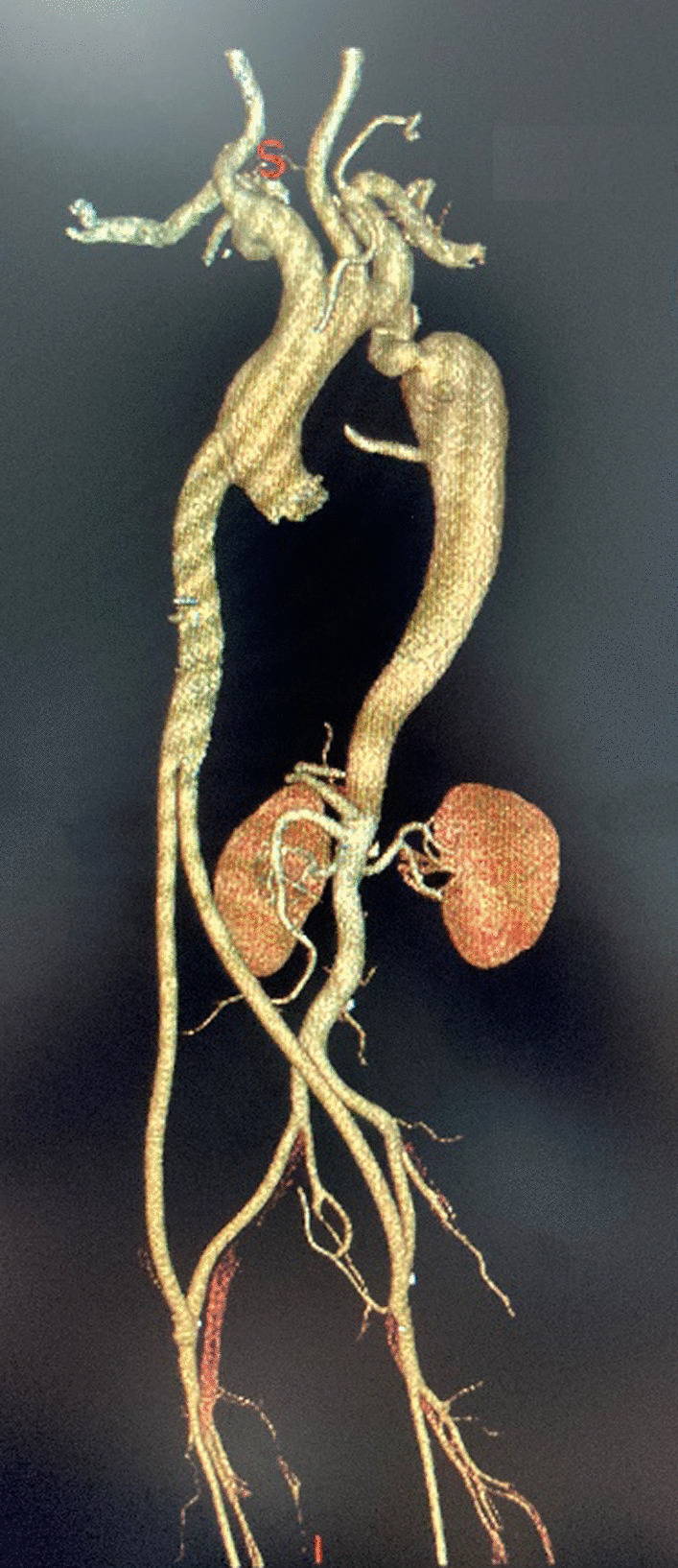
Postoperative CTA of AFB

The patient was placed in a supine position and invasive upper and lower limb BPs were monitored. First, bilateral femoral arteries were dissected through groin incisions (longitudinal or transverse). Second, repair procedure for the concomitant cardiac anomaly was conducted through median sternotomy. The arterial cannulation sites were selected as follow: femoral artery was cannulated for lower body perfusion and ascending aorta or right axillary artery (when ascending aorta should be replaced) was cannulated for upper body perfusion. After the concomitant cardiac procedure was completed, a Y-shaped prosthetic conduit (MAQUET, polytetrafluoroethylene, diameter 16-9 mm or 18-9 mm) was delivered through the tunnel in peritoneal cavity from mediastinum toward the groin incision using a smooth-ended tunnelizer. The prosthetic conduit was first anastomosed to the ascending aorta by end-to-side after the ascending aorta was partially clamped. The conduit was then anastomosed to both femoral arteries (Fig. 2).

#### Hybrid strategy

The position and monitor of BPs were the same as the AFB strategy. All the patients had the retrograde femoral artery catheterization. After advancing a flexible guidewire across the coarctation, a pigtail catheter was advanced over the wire into the aortic arch. In those with severe coarctation, graded dilatation by progressively larger balloons was performed. The balloon size for pre-dilation was adjusted based on the coarctation anatomy but not more than 5 times the stenosis diameter. The stent diameter was selected to be 20–30% greater than the diameter of the normal aorta at the level of distal arch. The stent length was selected to ensure that the stenosed segment was in the middle of the stent. After deployment of the stent, post-dilation was performed with a balloon in some patients with a pressure gradient of more than 5 mmHg. The maximum diameter of the balloon was selected not more than the diameter of the descending thoracic aorta at the level of the diaphragm. Finally, angiography was repeated and the pressure was recorded across the stented segment. The cardiac procedure was then performed through median sternotomy. Detailed information of all stents and balloons used was showed in Additional file 1: Table S1.

### Follow up

Patients were followed up with a mailed questionnaire or telephone call, by contacting the referring cardiologist or general practitioner. We defined PSG > 20 mmHg and (or) imaging (CTA or ultrasound) findings of significant stenosis (or occlusion) in the stent or artificial graft as the positive event, considering that PSG > 20 mmHg is a recognized interventional indication of COA [17].

### Statistic analysis

Categorical variables were reported as frequency (percentage %), and were compared between groups with the chi-squared test or Fisher exact test. Shapiro–wilk test was adopted for normal distribution analysis. Continuous variables were reported as mean ± SD or as median with interquartile range(as median [IQR]), and were compared with the two-sample *t* test (paired *t* test for the normal distribution data collected at different time points from the same group) or the Wilcoxon rank sum test as appropriate. *P* value of less than 0.05 was considered statistically significant. R software version 4.0.2 was used for statistical analysis. Graphpad Prism for Windows version 6.0 was used to create graphs.

## Results

50 patients were enrolled in this study, and were separated into AFB group (n = 30) or hybrid group (n = 20). All the cases were technical success. The baseline clinical data was listed in Table 1. The mean age of the AFB group was 39.5 ± 14.0 years, older than the hybrid group (27.9 ± 8.5 years, *P* = 0.002). The median diameter of the narrowest COA segment was 4.0 mm for the AFB group and 4.5 mm for the hybrid group (*P* = 0.218). The ratio of the narrowest COA dimension (mm) to the dimension of the normal descending aorta at diaphragm level (mm) in the AFB group was 0.15, whereas the ratio was 0.22 in the hybrid group (*P* = 0.038). The mean right upper limb systolic blood pressure (SBP) of the whole cohort was 155 ± 27.2 mmHg, 159.4 ± 28.1 mmHg for the AFB group and 148.4 ± 24.2 mmHg for the hybrid group, respectively(*P* = 0.153). The median peak systolic gradient (PSG) which was defined as upper limb SBP minus lower limb SBP, was 58.5 mmHg in total, and 68.0 mmHg for the AFB group and 46.5 mmHg for the hybrid group (*P* = 0.073). The mean upper-lower limbs blood gradient pressure was 21.4 ± 7.9 mmHg in total, 21.7 ± 8.2 mmHg for the AFB group and 21.0 ± 7.8 mmHg for the hybrid group (*P* = 0.744). The concomitant cardiac surgical procedures were summarized in Table 2.

**Table 1:** Main clinical characteristics of patients

Variables	Overall (n = 50)	Surgical group (n = 30)	Hybrid group (n = 20)	*P* value
Female, N (%)	16 (32.0)	9 (30.0)	7 (35.0)	0.951
Age (years), mean ± SD	34.9 ± 13.3	39.5 ± 14.0	27.9 ± 8.5	0.002**
BSA (m^2^), mean ± SD	1.8 ± 0.2	1.8 ± 0.2	1.7 ± 0.2	0.348
BMI (kg/m^2^), mean ± SD	21.7 ± 3.8	22.5 ± 3.8	20.5 ± 3.7	0.062
Previous stroke, N (%)	3 (6.0)	3 (10.0)	0 (0.0)	0.395
Tobacco use, N (%)	13 (26.0)	8 (26.7)	5 (25.0)	1
LVEF (%), median [IQR]	63.0 [59.3, 67.8]	61.5 [58.0, 67.0]	65.0 [62.5, 68.3]	0.187
Baseline upper-limb systolic blood pressure (mmHg), mean ± SD	155 ± 27.2	159.4 ± 28.1	148.4 ± 24.2	0.153
PSG baseline(mmHg), median [IQR]	58.5 [40.0, 75.5]	68.0 [41.5, 78.6]	46.5 [36.8, 67.0]	0.073
Mean upper-lower limb blood gradient pressure (mmHg), mean ± SD	21.4 ± 7.9	21.7 ± 8.2	21.0 ± 7.8	0.744
ABI, mean ± SD	0.63 ± 0.12	0.60 ± 0.13	0.66 ± 0.1	0.066
Diameter of narrowest aorta/Diameter of normal aorta(%), median [IQR]	16 [9, 27]	15 [6, 20]	22 [12, 33]	0.038*
Diameter of narrowest aorta(mm), median [IQR]	4.0 [2.4, 7.9]	4.0 [2.0, 7.6]	4.5 [3.0, 9.1]	0.218
Coarctation measurement, mm/BSA, median [IQR]	2.5 [1.3, 4.5]	2.4 [1.1, 4.2]	2.8 [1.6, 5.1]	0.177

*BSA* body surface area, *BMI* body mass index, *CPB* cardio-pulmonary bypass, *PSG baseline*: baseline peak systolic gradient, *IQR* interquartile range, *SD* standard deviation, *ABI* ankle brachial index

**Table 2:** Concomitant cardiac surgery

Concomitant cardiac surgery	Surgical (n = 30)	Hybrid (n = 20)	Total (n = 50)
Bentall + MVR	3	1	4
Bentall	8	3	11
Bentall + TAR	1	1	2
Wheat’s	1	1	2
CABG	3	0	3
AVR	7	7	14
MVR	0	1	1
MVR + AVR	1	0	1
MVP	0	3	3
VSD repair	3	3	6
Others	3	0	3

*MVR* mitral valve replacement; *TAR* total arch replacement; *CABG* coronary artery bypass grafting; *AVR* aortic valve replacement; *MVP* mitral valve plasty; *VSD* ventricular septal defect

The immediate operative outcomes were listed in Table 3. No perioperative death or severe complications were observed before discharge. There was no difference between groups in terms of the degree of SBP decline (*P* = 0.095, paired *T*-test). In addition, the PSG (*P* = 0.095, paired *T*-test), mean upper-lower limbs blood pressure gradient (*P* = 0.105, paired *T*-test) and ankle brachial index (ABI) (*P* = 0.167, paired *T*-test) were apparently improved without differences between groups. (Fig. 1) With respect to ICU stay and postoperative hospital stay, there were no differences between groups. (Table 3).

**Table 3:** Perioperative outcomes of patients

Variables	Overall (n = 50)	Surgical group (n = 30)	Hybrid group (n = 20)	*P* value
CPB time (minutes), mean ± SD	96.9 ± 47.8	105.8 ± 49.6	83.7 ± 42.9	0.108
ventilation time (hours), median [IQR]	15.5 [13.0, 18.0]	17.0 [13.3, 20.0]	14.5 [12.8, 17.0]	0.121
Drainage (ml), median [IQR]	1,030 [726, 1836]	1560 [970, 2220]	727 [577, 1020]	< 0.001***
Drains in situ (days), median [IQR]	5 [4, 6]	6 [4, 8.75]	4 [3.75, 5]	0.01*
Upper-limb systolic blood pressure (mmHg), mean ± SD	116.4 ± 12.2	119.7 ± 11.5	111.6 ± 11.5	0.022*
PSG post (mmHg), median [IQR]	10.0 [3.0, 14.0]	10.0 [4.0, 16.25]	10.0 [2.25, 13.25]	0.555
ΔPSG (mmHg) †, median [IQR]	48.0 [27.0, 65.5]	52.5 [37.0, 65.5]	32.5 [24.5, 61.5]	0.09
Mean upper-lower limb blood gradient pressure (mmHg), median [IQR]	5.0 [1.25, 7.22]	5.9 [4.1, 8.8]	2.7 [0.2, 6.7]	0.104
ABI	0.92 ± 0.10	0.92 ± 0.11	0.93 ± 0.1	0.666
ICU days, mean ± SD	2 ± 1.3	2 ± 1.3	2 ± 1.2	0.939
postoperative hospital stay (days), mean ± SD	9.0 ± 3.6	9.5 ± 4.1	8.1 ± 2.0	0.16

*IQR* interquartile range, *SD* standard deviation, *PSG post* postoperative peak systolic gradient, *ABI* ankle brachial index, *ICU* intensive care unit

†ΔPSG = (baseline PSG) − (postoperative PSG)

The follow-up outcomes were listed in Table 4. The mean follow-up time was 80.17 ± 19.6 months (48–112 months). There were 4 late deaths which were excluded from the analysis of the follow-up data. All these 4 late deaths were in the AFB group, but were not related to graft, COA or COA-related complications (for examples, cerebral hemorrhage caused by hypertension). One died of gastrointestinal bleeding 1 year after surgery, one died of pulmonary embolism 6 years after surgery and the other two died of heart failure caused by mechanical valve dysfunction 3 and 6 years after surgery. All the survivors (n = 46) had no COA related symptoms (lower limb ischemia or hypertension related symptoms). There were 2 cases in the AFB group developing unilateral graft occlusion during the follow-up. However, the BPs of the affected lower limbs remained intact compared to the contralateral limbs, which was considered related to sufficient blood flow provided from the contralateral side. No graft infection was found throughout the follow-up despite that the graft conduits were intraperitoneal. The mean upper limb SBP was 130.8 ± 13.6 mmHg, 132.8 ± 12.1 mmHg for the AFB group and 127.6 ± 15.3 mmHg for the hybrid group (*P* = 0.264). The mean PSG was 13.4 ± 9.2 mmHg in total, 13.7 ± 8.9 mmHg for the AFB group and 12.9 ± 9.7 mmHg for the hybrid group (*P* = 0.776). The median upper-lower limbs blood gradient pressure was 8.2 mmHg in total, 9.2 mmHg for the AFB group and 8.0 mmHg for the hybrid group (*P* = 0.947).

**Table 4:** Follow-up outcomes of patients

Variables	Overall (n = 46)	Surgical group (n = 26)	Hybrid group (n = 20)	*P* value
Follow-up time(months), mean ± SD	80.17 ± 19.6	76.92 ± 18.7	85.4 ± 20.6	0.176
Regular CTA, N (%)	17 (37.0)	11 (42.3)	6 (30.0)	0.785
PSG > 20 mmHg or severe stenosis of the graft revealed by CTA or ultrasound, N (%)	9 (19.6)	5 (19.2)	4 (20.0)	1
Upper-limb systolic blood pressure (mmHg), mean ± SD	130.8 ± 13.6	132.8 ± 12.1	127.6 ± 15.3	0.264
PSG FU(mmHg), mean ± SD	13.4 ± 9.2	13.7 ± 8.9	12.9 ± 9.7	0.776
ΔPSG(mmHg) †, median [IQR]	46.0 [24.3, 61.8]	51.5 [27.0, 63.8]	36.5 [23.0, 52.0]	0.150
Mean upper-lower limb blood gradient pressure (mmHg), median [IQR]	8.2 [4.6, 14.0]	9.2 [5.18, 13.2]	8.0 [2.3, 14.3]	0.947
ABI, mean ± SD	0.9 ± 0.08	0.9 ± 0.08	0.91 ± 0.09	0.676

*IQR* interquartile range, *SD* standard deviation, *PSG FU* follow-up peak systolic gradient, *CTA* computed tomographic angiography, *ABI* ankle brachial index

†ΔPSG = (baseline PSG) − (last follow-up PSG)

Blood pressure relevant parameters were displayed in Fig. 1. The improvement was pronounced in both groups and no difference was presented between groups.

## Discussion

COA concomitant with other cardiac anomalies requiring surgical intervention is associated with greater risk for correction, especially in adults. For a long time, a staged surgical strategy was proposed for these patients. The conventional staged strategy usually includes an anatomic correction of COA through thoracotomy and a cardiac surgery through sternotomy. The separated operations have some notable disadvantages: (1) The first-stage COA repair might produce fatal hemodynamic instability if the concomitant cardiac lesion is severe enough [7, 9]. (2) The traditional COA anatomical correction is prone to damage the recurrent laryngeal nerve and might suffer a terrible bleeding problem when the coarctation is long-standing with rich collateral circulation [5]. (3) Patients need to undergo two different incisions which lead to a greater trauma and heavier cost. Concerning the cumulative risks of staged strategy, one-stage strategy has been more popular in recent years. One-stage strategy for COA management usually involves one of the following techniques: extra-anatomic bypass from ascending aorta to abdominal aorta; intrapericardial ascending-descending aortic bypass and balloon plasty with stent implantation (hybrid surgery). Moreover, in current study, we innovatively adopted aortofemoral bypass (AFB) technique to deal with COA in one-stage strategy. Even though the ascending to descending or abdominal aorta bypass has become a standard manner in one-stage strategy and has been proved effective by some other studies [7, 9, 18], the AFB technique, also known as a manner of extra-anatomic bypass, outperform the traditional extra-anatomic techniques in following aspects: (1) Simple procedure: the distal anastomosis sites are at bilateral femoral arteries which are much easier to be exposed and dissected. (2) Relatively less or small incisions: no lateral thoracotomy or laparotomy is required. (3) Less abdominal complications: the AFB technique, unlike the traditional manner which may disturb abdominal organs when performing the distal anastomosis, seldom affects the abdominal organs. (4) Lower risk of distal anastomosis bleeding and easier hemostasis: the distal anastomoses of AFB have less probabilities to bleed due to lower tension. Even if there is bleeding, it is easy to be found and ended. On the contrary, the traditional manner could not provide a good access to the bleeding anastomosis. However, the main problem of AFB is the long-term patency of the graft vessels. Crawford et al. [19] reported a 3 years graft patency of 80% in aortoiliac occlusive patients treated with thoracofemoral bypass. In current study, occlusion occurred only in 2 cases (7.7%) during the follow-up. The long-term patency was promising.

The intrapericardial ascending-descending aortic bypass is an extra-anatomic approach that uses a shorter graft probably less prone to occlusions. However, in such method, the heart is retracted cephalad, which will affect hemodynamics and require the assistance of CPB, especially for those who need synchronous cardiac surgery. Furthermore, the exposure is especially difficult in obese patients or those with barrel-shaped thorax [9].

Anatomic correction with an interposition graft implanted via the median sternotomy is the optimal method for junior COA patients without previous interventions, even for those complicated with cardiac defects. However, in some adult COA patients, the lesions are often more troublesome: the vascular elasticity is reduced, and the collateral circulation is extremely rich. It is difficult to perform COA anatomical correction with synchronous cardiac surgery through midline incision. Consequently, a concomitant extra-anatomic bypass for COA may have a better prognosis. This study is the first to preliminarily assess the feasibility of AFB method for one-stage treatment of complicated COA. Due to the lack of experience and the very limited number of cases, no clear indications for AFB could be established.

Over the decades, the interventional stenting technology has achieved very fruitful clinical results in treatment of COA, [12, 14, 20, 21] and has become the treatment of first choice in adults in many centers [22]. However, the stents are not suitable for the coarctation located at the arch of which the curvature is too sharp. The reason could be an increased incidence of internal leakage outgrowth of the poor morphological structure match between stents and highly curved arch vessels [10, 17, 23]. Besides, in severe stenosis or arch hypoplasia cases, the stenting method is related to a poorer prognosis with a higher re-intervention rate [12]. Forbes et al. [16] reported a multicenter study with a large sample size (n = 217) on stenting for COA, suggesting that patients with restenosis after stenting and requiring re-intervention or surgery had an average baseline stenosis degree (ratio of coarctation dimension to the normal aortic dimension at diaphragm level) of 25% and an average postoperative stenosis degree of 66%, which was much lower than the mean postoperative degree (82%) of the whole group. He suggested that postoperative ratio of 60% appeared to be an important ratio regarding the requirement for reintervention [16]. Therefore, a patient with more severe stenosis would have a higher probability of reintervention after stenting. Similarly, we also found that in our study, the severity of stenosis was greater in the AFB group than the hybrid group (15% vs 22%, *p* = 0.038, see Table 1). Consequently, it is reasonable to consider AFB if the ratio is low. Furthermore, Forbes et al. [24] revealed a higher aortic wall injury rate in hypoplasia cases when treated with stenting. In summary, we suggested that the elder and hypoplasia patients should tend to the extra-anatomic strategy due to the calcification and reduced elasticity of the aortic wall tissue.

Compared with AFB strategy, the one-stage hybrid procedure has higher requirement on skills and facilities, needing a hybrid operation room and a hybrid team. Consequently, it is not easy to carry out in smaller cardiac centers. Otherwise, the hospitalization time and cost will be significantly increased if adopting a two-stage hybrid strategy instead.

Both of the AFB and hybrid strategy could significantly improve the BP relevant parameters (PSG, mean upper-lower limbs blood gradient pressure, upper limb SBP and ABI) after surgery (Fig. 1). No differences were found between groups, suggesting that the AFB technique had the similar effect on systemic BP compared with the hybrid (stenting) technique. Although 2 cases of unilateral graft occlusion were identified in AFB group during the follow-up, their homolateral lower limbs’ BPs were intact and no re-intervention indications were ascertained, proving that the contralateral bridge graft could provide sufficient blood flow to the occluded side.

The positive event (PSG > 20 mmHg or imaging findings of significant stenosis or occlusion in the stent or artificial graft) rates of two groups were almost the same (19.2% vs 20.0%, *p* = 1.0, Table 4). The hybrid strategy is undoubtedly irreplaceable, but pending further studies, it is reasonable to select the AFB strategy as an alternative in light of its promising outcomes.

### Limitations

In this study, follow-up results showed that the proportion of patients undergoing regular aortic CTA beyond one year after surgery was not high. A total of 17/46 patients in the whole group underwent aortic CTA one year after surgery, the AFB group had 11/26 patients and the hybrid group had 6/20 patients. The possible reason is that patients in our center came from across the whole country, but follow-up examinations were often conducted locally, the regional differences in medical resources and knowledge level led to an ignorance of routine aortic CTA exams in most inexperienced medical institutions.

### Future work

In this study, 2 cases in the AFB group had graft-related occlusion. Unfortunately, there is no guideline or consensus on anticoagulation methods for the artificial graft. Researches could be carried out on the anticoagulation strategy of AFB to better the management of such patients.

## Conclusion

The bilateral aortofemoral bypass surgery is a safe and effective method which can be used in one-staged surgical strategy to treat coarctation complicated by cardiac anomalies and can be an alternative to the hybrid method.

## Supplementary Information

**Additional file 1.** Detailed information of stents and balloons.

## Data Availability

The datasets used and/or analysed during the current study are available from the corresponding author on reasonable request.

## Abbreviations

COA: Coarctation
AFB: Aortofemoral bypass
CTA: Computed tomography angiography
BP: Blood pressure
SBP: Systolic blood pressure
PSG: Peak systolic gradient
ABI: Ankle brachial index
ICU: Intensive care unit
