# Supplementary material for: Outcomes of one-staged procedures to treat aortic coarctation complicated by cardiac anomalies
Source: BMC Cardiovasc Disord. 2022 Jul 3;22:302. doi: 10.1186/s12872-022-02739-x (PMC9250724; doi:10.1186/s12872-022-02739-x)
Supplement: Supplementary file 1 — Additional file 1. Detailed information of stents and balloons. [file 12872_2022_2739_MOESM1_ESM.docx]

| **Patient** | **Stent name** | **Stent product code** | **Stent length(mm)** | **Stent diameter(mm)** | **Balloon name** | **Balloon product code** | **Balloon diameter(mm)** | **Balloon length(mm)** |
| --- | --- | --- | --- | --- | --- | --- | --- | --- |
| 1 | NuMED CCP | CVRDCP8Z39 | 39 | 12-24 | NuMED BIB | BB017 | 20 | 45 |
| 2 | NuMED CCP | CVRDCP8Z34 | 34 | 12-24 | NuMED BIB | BB041 | 18 | 40 |
| 3 | NuMED CCP | CVRDCP8Z39 | 39 | 12-24 | NuMED BIB | BB017 | 20 | 45 |
| 4 | NuMED CCP | CVRDCP8Z39 | 39 | 12-24 | NuMED BIB | BB016 | 16 | 45 |
| 5 | NuMED CCP | CVRDCP8Z34 | 34 | 12-24 | NuMED BIB | BB014 | 20 | 40 |
| 6 | NuMED CCP | CVRDCP8Z34 | 34 | 12-24 | NuMED BIB | BB038 | 14 | 40 |
| 7 | NuMED CCP | CVRDCP8Z39 | 39 | 12-24 | NuMED BIB | BB041 | 18 | 40 |
| 8 | NuMED CCP | CVRDCP8Z39 | 39 | 12-24 | NuMED BIB | BB017 | 20 | 45 |
| 9 | NuMED CCP | CVRDCP8Z39 | 39 | 12-24 | NuMED BIB | BB016 | 16 | 45 |
| 10 | NuMED CCP | CVRDCP8Z34 | 34 | 12-24 | NuMED BIB | BB041 | 18 | 40 |
| 11 | NuMED CCP | CVRDCP8Z39 | 39 | 12-24 | NuMED BIB | BB064 | 22 | 45 |
| 12 | NuMED CCP | CVRDCP8Z39 | 39 | 12-24 | NuMED BIB | BB016 | 16 | 45 |
| 13 | NuMED CCP | CVRDCP8Z39 | 39 | 12-24 | NuMED BIB | BB041 | 18 | 40 |
| 14 | NuMED CCP | CVRDCP8Z39 | 39 | 12-24 | NuMED BIB | BB013 | 16 | 40 |
| 15 | NuMED CCP | CVRDCP8Z39 | 39 | 12-24 | NuMED BIB | BB017 | 20 | 45 |
| 16 | NuMED CCP | CVRDCP8Z34 | 34 | 12-24 | NuMED BIB | BB041 | 18 | 40 |
| 17 | Medtronic Endurant | ENEW2828C80EE | 80 | 28 | NuMED BIB | BB014 | 20 | 40 |
| 18 | Medtronic Endurant | ENEW2828C80EE | 80 | 28 | NuMED BIB | BB014 | 20 | 40 |
| 19 | NuMED CCP | CVRDCP8Z34 | 34 | 12-24 | NuMED BIB | BB029 | 18 | 45 |
| 20 | NuMED CCP | CVRDCP8Z45 | 45 | 12-24 | NuMED BIB | BB064 | 22 | 45 |

Supplement table 1. Detailed information of stents and balloons
